# Modelling Extraction of White Tea Polyphenols: The Influence of Temperature and Ethanol Concentration

**DOI:** 10.3390/antiox3040684

**Published:** 2014-10-21

**Authors:** Sara Peiró, Michael H. Gordon, Mónica Blanco, Francisca Pérez-Llamas, Francisco Segovia, María Pilar Almajano

**Affiliations:** 1 Department of Health Microbiology and Parasitology, Faculty of Pharmacy, Barcelona University, Avenue Joan XXIII s/n, 08028 Barcelona, Spain; E-Mail: sara.peiro@yahoo.es; 2 IRIS-Innovació I Recerca Industrial i Sostenible, Avda. Carl Friedrich Gauss nº11, 08860 Barcelona, Spain; 3 Chemical Engineering Department, Technical University of Catalonia, Avda Diagonal 647, 08028 Barcelona, Spain; E-Mail: segoviafj@gmail.com; 4 Department of Food and Nutritional Science, University of Reading, Whiteknights, P.O. Box 226, Reading RG6 6AP, UK; E-Mail: m.h.gordon@reading.ac.uk; 5 Department of Applied Mathematics III, Technical University of Catalonia, ESAB, Campus del Baix Llobregat, Esteve Terradas 8, 08860 Barcelona, Spain; E-Mail: monica.blanco@upc.edu; 6 Department of Physiology and Pharmacology, School of Biology, University of Murcia, Campus de Espinardo, 30100 Murcia, Spain; E-Mail: frapella@um.es

**Keywords:** white tea, polyphenols, extraction, antioxidant, RSM, MECK

## Abstract

The optimization of the extraction of natural antioxidants from white tea has fostered intensive research. This study has investigated the effects of ethanol-water mixtures, temperature and time on the extraction of polyphenols and antioxidant components from white tea. The response surface methodology was applied to identify the best extraction conditions. The best conditions to maximize the extraction of total polyphenols were: ethanol, 50%, for 47.5 min. Although the yield of polyphenols was optimal at 65 °C, the maximum antioxidant capacity was achieved with an extraction temperature of 90 °C. This study has identified the optimal conditions for the extraction of tea liquor with the best antioxidant properties. Epigallocatechin gallate, epicatechin gallate, epigallocatechin and epicatechin were extracted from white tea at concentrations up to 29.6 ± 10.6, 5.40 ± 2.09, 5.04 ± 0.20 and 2.48 ± 1.10 mg/100 g.

## 1. Introduction

Natural antioxidants are increasingly appreciated by consumers due to both their inherent positive effects [1] and to the possibility of using them as a source of natural additives to replace synthetic ones [2,3,4]. Tea is a natural plant that is a rich source of natural antioxidants and provides a high free radical scavenger activity [5,6].

Tea, the infusion from *Camellia sinensis* (L), is one of the world’s most widely consumed beverages [7,8]. Its medicinal properties have been widely explored [9]. In addition, its health promoting properties have been known from the early periods of the Chinese civilization, going back almost 5000 years [10].

Teas vary in properties depending on geographical origin, climatic conditions and processing methods [7], but in general, they can be classified into three types: unfermented (green and white teas), partially fermented (oolong tea) and completely fermented (black tea) [9,11]. White tea is the least processed tea. It is exclusively prepared from very young tea leaves and buds, which are harvested before being fully open and are processed by air drying [10]. This less extended processing confers to white tea its special and highly appreciated odour and flavour characteristics [12,13].

Some *in vitro* studies have reported and characterised the antioxidant activity of white tea [11,14,15]. Other studies have focused on its protective effects on live cells subject to induced oxidative stress [16,17,18,19,20]. Some of them have concluded that white tea extract is a neuroprotector, because it reduces the effect of hydrogen peroxide on cells [6]. This could be relevant to enhance the protection against neurodegenerative diseases, such as Alzheimer’s or Parkinson’s. Recent studies [21] suggest that the protective action of white tea in oxidative stress *in vitro* is related to the maintenance of the normal redox status of cells when they are susceptible to damage by free radicals.

The antioxidant capacity of white tea extracts, which can be measured *in vitro* by various assays, including oxygen radical absorbance capacity (ORAC), Trolox equivalent antioxidant capacity (TEAC), ferric reducing antioxidant power (FRAP) or diphenylpicrylhydrazyl assay (DPPH), among others [22,23,24]. This activity is linked to the high content of flavan-3-ols, which are also known as catechins. The major catechins present in tea are: epigallocatechin (EGC), catechin (C), epigallocatechin gallate (EGCG), epicatechin (EC) and epicatechin gallate (ECG) [8,13,25].

These catechins have a high health benefits and industrial interest. They can be used in the pharmaceutical, cosmetic and food industries as a source of additives or as a source of antioxidants for functional foods [4,9]. As a result of these benefits, more effective extracts are a research focus of interest.

The main goal of this contribution is to determine the optimal extraction conditions of the main antioxidant compounds from white tea. In this study, the response surface methodology (RSM) was used for the optimization of extraction variables (time, temperature and % of ethanol) to enhance the yield of polyphenols and antioxidant activity [15,26,27].

## 2. Experimental Section

### 2.1. Chemicals, Reagents and Equipment

Methanol, ethanol, acetone, sodium carbonate, Folin-Ciocalteu reagent, sodium tetraborate, sodium phosphate dibasic and sodium hydroxide were of analytical grade from Panreac (Barcelona, Spain). Gallic acid (GA), rutin, 6-hydroxy-2,5,7,8-tetramethylchroman-2-carboxylic acid (Trolox), 2,4,6-tris (1-pyridyl)-5-triazine (TPTZ), phosphate buffered saline, ferric chloride, potassium persulfate, Tween 20, 2,2′-azino-bis(3-ethylbenzothiazoline-6-sulfonic acid) diammonium salt (ABTS), sodium dodecyl sulphate (SDS), (+)-catechin (C), (−)-epicatechin (EC), (−)-epigallocatechin (EGC), (−)-epicatechin gallate (ECG), (−)-epigallocatechin gallate (EGCG) and caffeine were purchased from Sigma-Aldrich Company Ltd. (Gillingham, UK). Ultrapure water obtained from a Milli-Q system from Millipore (Milford, MA, USA) was used throughout. Spectrophotometric measurements were taken on a Perkin Elmer FTIR spectrometer (Perkin Elmer, Paris, France). Fluorometric measurements were taken with a Florestar omega fluorimeter. Micellar electrokinetic chromatography (MECK) was carried out using the Packard 3DCE capillary electrophoresis system equipped with a diode-array detector from Agilent (Agilent Technologies, Santa Clara, CA, USA).

### 2.2. Tea Samples and Preparation

White tea was purchased from Manantial de Salud (Herbocat SL, Barcelona, Spain) and was stored at room temperature (22 ± 2 °C) in a desiccator. The samples were extracted by infusion.

Infusions were filtered through Whatman paper filters n.2 (GE Healthcare, Amersham Place, UK). All white tea extracts were protected from light and stored at −20 °C until needed.

### 2.3. Determination of Total Phenolic Content

The total phenol (TP) content of each extract was determined in duplicate by the Folin-Ciocalteu method according to Almajano *et al.* [16]. The mixture was allowed to stand in the dark at room temperature for 1 h and was finally diluted to an appropriate final volume with distilled water. Absorbance was measured at 765 nm against a blank containing distilled water instead of extract. Values were determined from a calibration curve prepared with gallic acid standard (GAE) (2–14 mg·L^−1^ final concentration) and rutin (1.25–15 mg·L^−1^ final concentration). Results are expressed as mg of gallic acid equivalents g^−1^ of dry weight (mg GAE/g DW) or mg of rutin equivalents/g of dry weight (mg rutin equivalents/g DW), respectively.

### 2.4. Micellar Electrokinetic Capillary Chromatography (MECK)

MECK is a separation mode of capillary electrophoresis (CE), which can be applied in the separation of neutral and charged compounds. The principle of separation is based on the differential migration of the ionic micelles and the bulk running buffer under electrophoresis conditions and on the interaction between the analyte and the micelle. The micelle is prepared by adding a surfactant, generally sodium dodecyl sulphate (SDS), into the running buffer. MECK is suitable for separation and quantification of natural antioxidants, because it is rapid, gives an efficient separation, uses minimum amounts of sample and reagents and is low cost [17,25,27,28,29].

MECK was carried out with the diode-array detector set at 200 and 260 nm to detect catechins and gallic acid simultaneously. A fused-silica capillary, with extended light path, 50μm i.d., 34 cm total length and 25.5 cm effective length, was used for the separation. The separation voltage was kept at 30 kV with an intensity of 35 μA. The temperature of the capillary was set to 21 °C and controlled with the help of a thermostat. The capillary was conditioned with NaOH (0.1 M, 10 min) followed by washing with an abundant flow of Milli-Q water and then of the buffer solution (155 min). Buffer (pH 7) was prepared daily with 5 mM sodium tetraborate, 60 mM sodium phosphate dibasic and 50 mM SDS.

All standard solutions (C, EC, GC, EG, EGCG) and sample extracts were injected in triplicate. A calibration curve prepared with standards was used to quantify the components (mg/L).

### 2.5. Determination of Antioxidant Activity

#### 2.5.1. Trolox Equivalent Antioxidant Capacity (TEAC)

The method used was based on Re *et al.* [30]. 2,2′-Azino-bis(3-ethylbenzothiazoline-6-sulfonic acid) diammonium salt (ABTS, 7 mM) and potassium persulfate (2.45 mM, final concentration) were dissolved separately in water, and then, the mixture was made up to volume in a 10 mL volumetric flask. The mixed solution was transferred to an amber bottle, covered with aluminium foil and allowed to stand at room temperature (RT) for 12–16 h in the dark. The ABTS ^•+^ solution was diluted with phosphate buffered saline (PBS) (pH 7.4, 1:100) and equilibrated at 30 °C, to an absorbance of 0.7 ± 0.02) at 734 nm, read in a Hewlett Packard 8452A diode array spectrophotometer (WaldBronn, Germany). An appropriate dilution of the extract was added to ABTS^+^ solution in the proportion of 1:100. PBS (pH 7.4) was used as the blank. After mixing, the absorbance at 734 nm was measured immediately and, then, every minute for 5 min. Duplicate determinations were made for triplicate extractions. The percentage inhibition was calculated from the absorbance values at 5 min.

The relative change in sample absorbance was calculated according to the following equation:

(1) $$\text{Δ}A_{sample}=\frac{A_{t=0(sample)}-A_{t=5(sample)}}{A_{t=0(sample)}}-\frac{A_{t=0(solvent)}-A_{t=5(solvent)}}{A_{t=0(solvent)}}$$

The TEAC value was determined from a Trolox calibration curve (ranging from 1 to 10 μM final concentration). Results are expressed as μmol of Trolox/g of DW.

#### 2.5.2. Oxygen Radical Antioxidant Capacity (ORAC)

The ORAC method [28,31] is widely used in food science and biology. A stock solution of fluorescein (FL) was prepared by dissolving 2 mg of FL in 100 mL of phosphate buffer (PBS) 75 mM and pH 7. The stock solution was stored under refrigeration in the dark. The working FL solution (78 nM) was prepared daily by adequate stock dilution in PBS. The 2,2′-Azobis(2-methylpropionamidine) dihydrochloride (AAPH)radical solution (221 mM) was prepared daily by dilution in PBS. The standard used was Trolox solution.

The ORAC values were calculated using a regression equation between the Trolox concentration and the net area of the fluorescence decay curve (area under curve, AUC). ORAC was expressed as μM Trolox equivalents (μM TE) and was calculated by applying the formula in Equation 2:

(2) $$AUC=\left(0.5+\left(\sum_{i=1}^{i=31}\frac{f_{i}}{f_{1}}\right)\right)\cdot CT$$

where: *i*, the number of cycles; *f*, florescence units; *CT*, time of each cycle in minutes; in this case, *CT* is 2 min.

### 2.6. Statistical Analysis

The results obtained were analysed statistically using Minitab 5.1 for Windows (Minitab Inc., State Collage, PA, USA) and expressed as the means ± standard deviations. Any significant difference between solvents and samples was determined by one-way analysis of variance (ANOVA, considering significant differences at *p* < 0.05.

### 2.7. Response Surface Methodology (RSM)

The data were modelled and analysed by RSM, which is a collection of mathematical and statistical techniques suitable for problems in which a response of interest is influenced by several variables. It uses quantitative data from appropriate experimental designs to model and optimize the combination of factors that yield a desired response near the optimum [26].

In order to determine the optimum conditions for the extraction of tea polyphenols and to evaluated their activity with TEAC and ORAC, assays were performed using low and high levels for the independent variables, EtOH (%), time (min) and temperature (°C), in accordance with a factorial experimental design. The results of the preliminary trials of a two-level, three-variable full factorial design were taken into account, which involved three replicated runs. A two-level, three-factor and central composite design was chosen for this experimental design using the Minitab package for Windows software (Minitab, State Collage, PA, USA). Coded levels for independent variables are presented in Table 1.

**Table 1:** Variables and ranges used on the experimental design for the study of temperature (*T*), time (*t*) and % ethanol.

Variables	Range and Level		
	1	0	1
Temperature (°C)	40	65	90
Time (min)	5	47.5	90
% EtOH	0	50	100

Coefficients of the full model were evaluated by regression analysis and tested for their significance. The non-significant coefficients (*p* > 0.05) were eliminated on the basis of *p*-values after examining the coefficients, and the models were finally refined. For the first-order model, we considered a 2^3^ factorial design augmented by three centre points. For the second-order model, we augmented the design with six points (star design). From the values (displayed in Table 1) and assuming a second order polynomial model, at least 17 experiments must be carried out to solve the matrix and the error evaluation. The resulting factorial central composite design for the two-level and three-factor scheme with 17 treatments in total is described in Table 2. The response surface values are the concentrations of resulting tea polyphenols, which are shown in the results section.

## 3. Results and Discussion

### 3.1. Selection of Extraction Solvents

It is reported by other researchers [26,32,33] that ethanol and methanol were effective solvents for extracting phenolic compounds. For this study, only ethanol was used, since it is food-grade and also cheaper than methanol.

### 3.2. Experimental Design

The experimental design was carried out to evaluate the effects of temperature, solvent concentration and time on antioxidant extraction. The variables and ranges used are shown in Table 1. The *p*-value for each term analysed in each parameter is shown in Table 2. The initial model and the final reducted model are shown in Table 3. The final reducted model, with all statistically significant terms, has a higher predicted *R*^2^, which means that it is more reliable in estimating a response.

TP, ORAC, TEAC and caffeine show high number interactions with statistical significant terms (*p* < 0.05), and a good adjustment was obtained.

### 3.3. Total Polyphenol Content

The extraction efficiency of different concentrations of aqueous ethanol, temperature and time for the extraction of total polyphenols from white tea leaves was investigated using a central composite design. The total phenolic content in the white tea extracts ranged from 20.93 to 178.70 mg as GAE/g tea; see Table 2. It was observed that the best yield occurred with 47.5 min of extraction at 65 °C, using 48% ethanol.

All of the linear coefficients and two quadratic coefficients (EtOH^2^ and *t*^2^) were significant. The final response model (using uncoded units) to predict the yield of tea polyphenols is shown in Equation 3:

(3) $$TP=24.1036+2.3871\;EtOH+1.7821\;t+0.6350\;T-0.0270\;EtOH^{2}-0.0156\;t^{2}$$

where *TP* is the response variable and *EtOH*, *t* and *T* are the values of the independent variables, namely the concentration of ethanol, extraction time and temperature, respectively.

**Table 2:** Experimental design obtained and the experimental values obtained from the determination of total polyphenols, antioxidant capacity of the extracts assessed by TEAC and ORAC and polyphenol content.

	Assay nº	% EtOH	*t* (min)	*T* (°C)	Experimental Values							
					TP	TEAC	ORAC	Caffeine	EGC	EGCG	ECG	EC
2^3^ factorial design	1	0	5	40	62.4 ± 3.1	596 ± 27	710 ± 83	11.31 ± 6.34	1.19 ± 0.53	3.22 ± 1.97	0.46 ± 0.26	0.55 ± 0.37
	2	96	5	40	20.9 ± 2.2	209 ± 23	437 ± 102	2.05 ± 0.43	1.86 ± 0.05	4.97 ± 0.44	1.08 ± 0.08	0.00 ± 0.00
	3	0	90	40	95.7 ± 4.1	945 ± 27	1396 ± 146	21.08 ± 0.90	4.00 ± 1.24	9.97 ± 3.58	0.97 ± 0.40	1.86 ± 0.53
	4	96	90	40	36.5 ± 8.6	348 ± 92	718 ± 156	3.68 ± 1.42	3.42 ± 1.83	10.50 ± 5.76	2.58 ± 1.36	0.33 ± 0.31
	5	0	5	90	96.1 ± 3.5	1067 ± 60	1053 ± 213	28.16 ± 2.13	0.64 ± 0.68	1.92 ± 1.04	0.30 ± 0.06	0.14 ± 0.05
	6	96	5	90	31.9 ± 2.9	341 ± 32	548 ± 177	3.52 ± 0.28	1.38 ± 0.21	1.21 ± 0.78	0.28 ± 0.17	0.22 ± 0.06
	7	0	90	90	112.6 ± 6.6	1165 ± 132	1355 ± 134	27.84 ± 1.88	1.16 ± 0.61	3.55 ± 2.44	0.64 ± 0.55	0.45 ± 0.45
	8	96	90	90	104.8 ± 2.1	1083 ± 50	1405 ± 45	9.18 ± 0.39	5.04 ± 0.20	10.90 ± 4.97	2.17 ± 0.93	1.29 ± 0.17
star design	9	0	47.5	65	94.8 ± 7.9	1042 ± 82	1244 ± 60	22.72 ± 2.26	0.80 ± 0.68	1.60 ± 0.76	0.18 ± 0.08	0.18 ± 0.20
	10	96	47.5	65	113.3 ± 24.9	529 ± 126	906 ± 285	3.49 ± 0.83	4.79 ± 2.28	18.73 ± 12.90	4.66 ± 3.15	0.68 ± 0.34
	11	48	5	65	148.5 ± 6.8	1777 ± 25	1914 ± 171	27.84 ± 0.82	1.71 ± 1.60	7.81 ± 6.41	1.25 ± 1.07	0.20 ± 0.34
	12	48	90	65	138.1 ± 25.6	1433 ± 221	1749 ± 296	20.36 ± 3.38	1.06 ± 0.32	5.88 ± 1.91	0.90 ± 0.43	0.15 ± 0.12
	13	48	47.5	40	144.4 ± 16.0	1466 ± 124	2387 ± 467	23.08 ± 1.73	2.01 ± 0.52	10.05 ± 3.88	1.71 ± 0.89	0.44 ± 0.19
	14	48	47.5	90	173.4 ± 7.9	1940 ± 117	2174 ± 245	26.66 ± 0.64	4.01 ± 0.20	19.51 ± 2.59	3.20 ± 0.44	1.28 ± 0.42
central design	15	48	47.5	65	163.3 ± 13.8	1674 ± 130	1811 ± 105	25.08 ± 0.11	3.96 ± 0.72	21.25 ± 6.78	3.58 ± 1.02	1.77 ± 0.40
	16	48	47.5	65	164.9 ± 7.9	1745 ± 130	1936 ± 205	26.85 ± 1.72	5.03 ± 1.06	27.45 ± 7.87	4.83 ± 1.26	1.31 ± 0.20
	17	48	47.5	65	178.7 ± 5.2	1820 ± 60	2132 ± 342	27.48 ± 2.37	4.71 ± 1.23	29.60 ± 10.60	5.40 ± 2.09	2.48 ± 1.10

Results are expressed as the mean of three replicates ± standard deviation; TP, total polyphenols (mg gallic acid equivalents (GAE)/g); TEAC, Trolox equivalent antioxidant capacity (μM TE/g); ORAC, oxygen radical antioxidant capacity (μM Trolox equivalents (TE)/g tea); Caffeine, epigallocatechin (EGC), epigallocatechin gallate (EGCG), epicatechin (EC) and epicatechin gallate (ECG) expressed as mg/g.

**Table 3:** The *p*-values for each of the constants in the equation of the mathematical model.

Term		*p*-Value							
		Response							
		TP	Caffeine	EGC	EGCG	ECG	EC	ORAC	TEAC
Complete Model	Constant	0.986	0.562	0.230	0.742	0.496	0.150	0.003	0.174
	% EtOH	0.000	0.000	0.000	0.118	0.000	0.690	0.000	0.000
	*t* (min)	0.008	0.156	0.002	0.007	0.003	0.007	0.170	0.416
	*T* (°C)	0.115	0.337	0.798	0.752	0.960	0.458	0.053	0.724
	% EtOH × % EtOH	0.000	0.000	0.610	0.045	0.786	0.270	0.000	0.000
	*t* (min) × *t* (min)	0.000	0.550	0.004	0.002	0.004	0.000	0.013	0.130
	*T* × *T*	0.157	0.959	0.470	0.717	0.550	0.563	0.049	0.958
	EtOH × *t* (min)	0.204	0.692	0.490	0.590	0.715	0.175	0.740	0.133
	% EtOH × *T* (°C)	0.344	0.004	0.720	0.730	0.209	0.269	0.276	0.538
	*t* × *T*	0.184	0.269	0.400	0.940	0.385	0.980	0.671	0.220
Reducted Model	Constant	0.038	0.003	0.027	0.344	−0.771	0.069	0.001	0.003
	% EtOH	0.000	0.000	0.017	0.060	0.016	-	0.000	0.000
	*t* (min)	0.000	0.000	0.096	0.000	0.130	0.041	0.006	0.000
	*T* (°C)	0.000	0.000	-	-	-	-	0.089	0.000
	% EtOH × % EtOH	0.000	0.000	-	0.018	-	-	0.000	0.000
	*t* (min) × *t* (min)	-	-	0.001	0.000	0.001	0.000	0.052	-
	*T* × *T*	0.000	-	-	-	-	-	-	-
	EtOH × *t* (min)	-	-	-	-	-	-	-	-
	% EtOH × *T* (°C)	-	0.003	-	-	-	-	-	-
	*t* × *T*	-	-	-	-	-	-	-	-

- : Term not applied in the Model

The coefficient of determination in a multiple regression equation measures the strength of the relationship between the independent variables and the (dependent) response. The value of the determination coefficient for the equation for tea polyphenols is *R*^2^ = 0.873, which indicates that only 13% of the total variation is not explained by the model.

**Figure 1:**
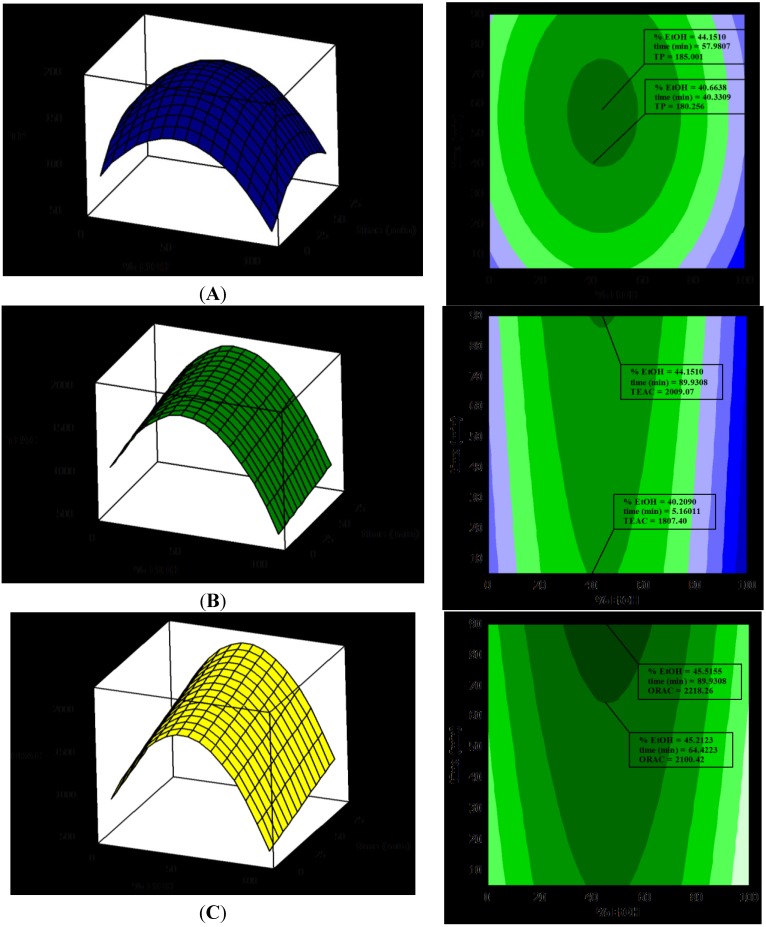
Response surface plots (**left**) and contour plots (**right**) showing the effect of ethanol concentration % in white tea extractions and the relations with antioxidant capacity evaluated by TP, TEAC and ORAC. (**A**) Response surface methodology (RSM) for TP and EtOH, (**B**) RSM for TEAC and ETOH and (**C**) RSM for ORAC and EtOH.

Focusing on the RSM (represented in Figure 1A), it can deduced that the TP dependency is quadratic, and the optimal results could be obtained by extracting with 45% ETOH for 57 min, at 90 °C.

The results of this study are different from those of other authors; Venditti *et al.* [10] obtained significantly higher values in white tea after steeping in cold water (RT) for two hours. This study found that extraction of polyphenols was poor for water and aqueous ethanol at 40 °C.

Acid pH is reported to improve polyphenol extraction [14,15,17,34]. Zimmerman [34] reported that at pH = 3, ECG extraction increased 20% and suggested that this may be due to two possible mechanisms. At a low pH, either the diffusion of flavonoids from the leaf into the aqueous phase increased or degradation of the leaf structure occurred, permitting better accessibility of the solvent to the leaf components. However, when lemon juice was used to reduce the pH, the antioxidant capacity of the lemon juice bioactive compounds was not considered [35,36].

### 3.4. Polyphenol and Caffeine Composition

Caffeine, EGC, EGCG, ECG, EC, catechin and gallic acid content were analysed by MECK. Catechin and gallic acid were not detected. All results obtained are shown in Table 2.

Caffeine shows a final response model (Equation 4) with good adjustment (*R*^2^ = 0.89) in the model.

Caffeine = 7.93192 + 0.41115*EtOH* + 0.21984*T* − 0.00481*EtOH*^2^ − 0.00166*EtOH* × *T* (4)

The caffeine extracted was in the range of 2.05–28.3 mg/g dry tea. The minimum values were observed in samples extracted with a high ethanol concentration. This is consistent with the low polarity of the caffeine molecule. There is no literature about the caffeine content of white tea samples.

EC, ECG and EG have a reduce number of terms and interactions with statistical significant *p*-values (Table 3). They have an equation with low *R*^2^. This fact suggested that the TP model responds to the synergic effect of all catechins contained in the sample.

Despite this bad adjustment, experimental results (Table 2) show that tea ECG content was in the range from 0.64 to 5.04 mg/g GAE. Lopez *et al.* [37], obtained similar ECG values (7.95 mg/g of tea) using an acid extraction method. Rusak *et al.* [13] reported a very high ECG content (42.3 mg/g of tea) after acid extraction and HPLC analysis. The latter author obtained 34.4 mg ECG/g dry tea using 40% ethanol for 30 min and 12.9 mg/g dry tea using 70% ethanol.

The EGC yield was 1.21–29.60 mg/g dry tea. The maximum yield was obtained with ethanol between 48% and 96% with an extraction time of 47.5 min and a temperature range of 56–90 °C. Rusak *et al.* [13] reported a range between 40.2 and 154 mg of EGC/g of dry white tea in bags and a range between 38.9 and 129 mg/g of EGC/g of dry tea in loose leaves analysed by HPLC. Lopez *et al.* [37] reported 11.1 mg EGC/g white tea, which is well within the range that was found by MECK analysis in this study.

The ECG content of the extracts corresponded to values between 0.18 and 5.40 mg ECG as GAE/g tea. Values reported by Lopez *et al.* [37] (3.19 mg/g dry tea) extracted from acidified samples were within this range. However, Rusak *et al.* [13] reported that 36.6 mg ECG as GAE/g dry white tea was extracted using 40% ethanol for a 15–30 min extraction time.

In EC analysis, a maximum extraction of 2.48 mg/g GAE was obtained in this study, which is similar to the value reported by Lopez *et al.* [37] (2.13 mg/g).

### 3.5. Antioxidant Capacity

The antioxidant activity of the different white tea infusions was assessed by the TEAC and ORAC assays. Some authors have shown significant differences in free radical scavenging activity according to the assay method used [23,38] and have reported that the TEAC assay is simpler and cheaper than the ORAC assay, but may give an underestimate of the antioxidant capacity. Tabart *et al.* [23] proposed that the mean of the values obtained using four different tests should be used.

A comparison between the two methods used in this study is presented, and the results are analysed using RSM.

#### 3.5.1. TEAC

The contour plot (Figure 1B) shows that the TEAC values were highest for EtOH concentrations between 40% to 50%. The extract with the maximum TEAC value was obtained with 43.7% EtOH for 90 min. Surface plots shows that there was a quadratic dependence on %EtOH and a linear dependence on extraction time. All of the linear coefficients were again significant, but just one quadratic coefficient (EtOH^2^) was included. The final response model (using uncoded units) to predict TEAC is shown in Equation 5:

(5) $$TEAC=325.540+33.821EtOH+2.314t+8.126T-0.384EtOH^{2}$$

where TEAC is the response variable. The value of the determination coefficient for the equation is *R*^2^ = 0.91 (only about 9% of the total variation is not explained by the model), so it can be concluded that there is a very good correlation between TEAC and the independent variables.

Samples 14 and 17, which had the highest TEAC values, had the highest TP values (Table 2). The polyphenols present were rich in EGCG, with moderate concentrations of ECG and EGC and a low concentration of EC (see Table 3). Salah *et al.* [39] reported that the relative antioxidant activity of the tea catechins assessed by the TEAC assay was in the order ECG > EGCG > EGC > EC, so it is clear that ECG and EGCG made important contributions to the antioxidant capacity.

#### 3.5.2. ORAC

For assessment of antioxidant capacity by the ORAC assay, only two linear coefficients (EtOH and *t*) and one quadratic coefficient (EtOH^2^) were significant. The final response model (using uncoded units) to predict ORAC is defined in Equation 6:

(6) $$ORAC=932.682+38.017EtOH+4.615t-0.415EtOH^{2}$$

where ORAC is the response variable. The value of the determination coefficient for the equation is *R*^2^ = 0.791, which indicates that less than 21% of the total variation was not explained by the model.

### 3.6. Correlation between TP, TEAC and ORAC

**Figure 2:**
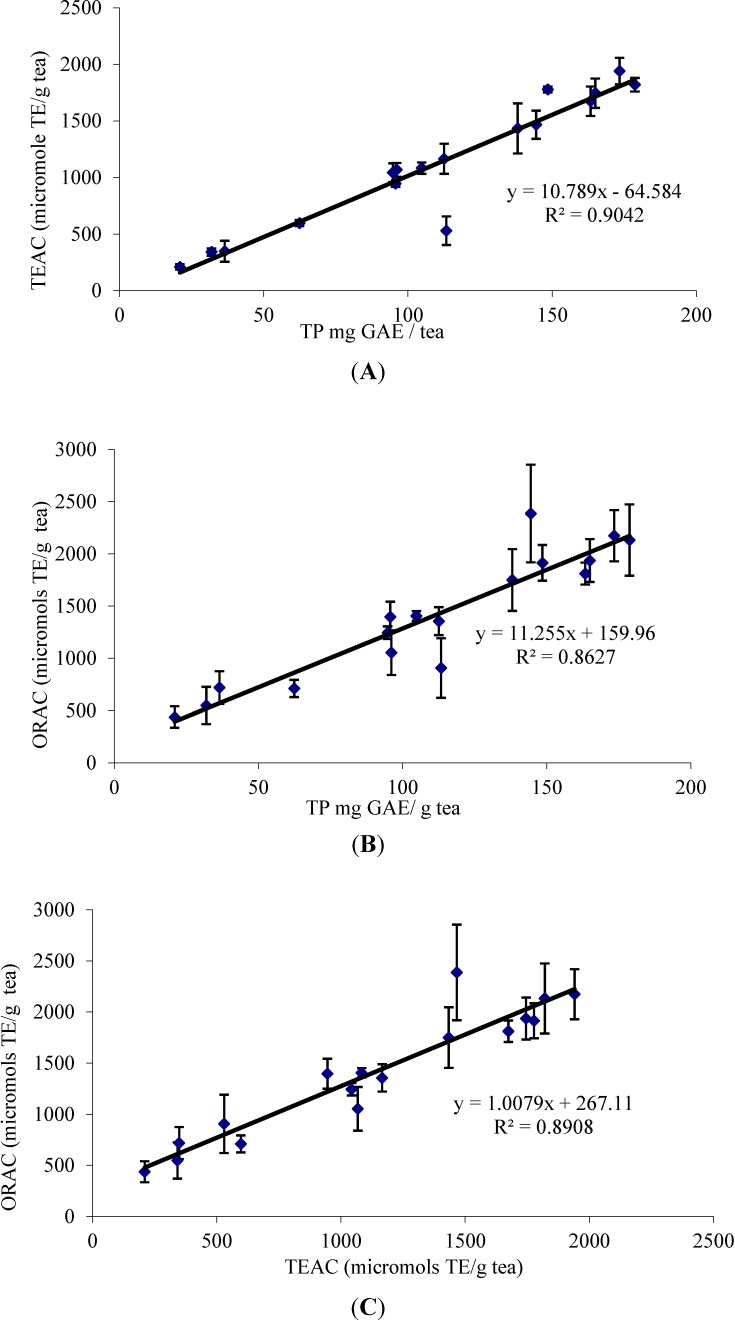
Correlation between TEAC, ORAC and total polyphenols (TP). (**A**) Correlation between TEAC and TP, (**B**) ORAC and TP and (**C**) ORAC and TEAC.

The study of the correlation between experimental data obtained in TP, TEAC and ORAC was also evaluated. Several authors have reported correlations between the results found for radical scavenging activity assessed by the TEAC and ORAC methods [39]. The results of this study show a correlation between TP, TEAC and ORAC values (Figure 2). The best correlation is that between total polyphenols (TP) and TEAC values (*R*^2^ = 0.90).

The correlation between TP and the ORAC values is not so good (*R*^2^ = 0.86). The most likely reason for this is that the TEAC assay only measures single electron transfer (SET) and can only measure the extent of inhibition by antioxidants. ORAC combines the time and magnitude of the inhibition, so the effects of slow reacting and fast reacting antioxidants differ in this assay, but not in the TP or TEAC assays.

All three variables in the extraction process had a significant effect on the polyphenols and TEAC value. EtOH and time had a much greater effect on the ORAC value, while temperature had less effect.

Figure 1 displays the surface plots of RSM, in which the fitted responses were plotted against changes in the factors, EtOH and time, whereas the temperature was held at three different levels (40 °C, 65 °C and 90 °C). EtOH and time are represented on the *x*-axis, while TP, TEAC and ORAC are shown on the *y*-axis.

The optimal conditions for the extraction of tea polyphenols predicted by the equation were: EtOH = 45%, *t* = 57 min and *T* = 90 °C. Likewise, the conditions for extracts with optimal TEAC values predicted by the equation were: EtOH = 40%, *t* = 90 min and *T* = 90 °C. Finally, the conditions for extracts with optimal ORAC values predicted by the equation were: EtOH = 40%–60% and *t* = 60 min, with no significant effect of temperature.

## 4. Conclusions

The highest amounts of polyphenols (both individually and as determined by the Folin-Ciocalteu assay) were extracted at intermediate values of the conditions studied (about 48% ethanol, 47.5 min and 65 °C). The TP values correlated with antioxidant capacity determined by both the ORAC and TEAC assays, although the best temperature for the extraction of radical scavenging components assessed by the TEAC assay was 90 °C. Time is the factor that was less important, in the ranges studied, and %EtOH had the greatest influence. This study has identified optimal conditions for the extraction of tea liquor with the best antioxidant properties.
